# CPEB2 enhances cell growth and angiogenesis by upregulating ARPC5 mRNA stability in multiple myeloma

**DOI:** 10.1186/s13018-023-03835-0

**Published:** 2023-05-26

**Authors:** Piaorong Zeng, Fujue Wang, Xingxing Long, Yixiong Cao, Feng Wen, Junjun Li, Zeyu Luo

**Affiliations:** The First Affiliated Hospital, Department of Hematology, Hengyang Medical School, University of South China, No.69, Chuanshan Road, Hengyang, 421001 Hunan Province People’s Republic of China

**Keywords:** Multiple myeloma, CPEB2, ARPC5, Cell growth, Angiogenesis

## Abstract

**Background:**

The process of multiple myeloma (MM) is the result of the combined action of multiple genes. This study aims to explore the role and mechanism of cytoplasmic polyadenylation element binding protein2 (CPEB2) in MM progression.

**Methods:**

The mRNA and protein expression levels of CPEB2 and actin-related protein 2/3 complex subunit 5 (ARPC5) were assessed by quantitative real-time PCR and western blot analysis. Cell function was determined by cell counting kit 8 assay, soft-agar colony formation assay, flow cytometry and tube formation assay. Fluorescent in situ hybridization assay was used to analyze the co-localization of CPEB2 and ARPC5 in MM cells. Actinomycin D treatment and cycloheximide chase assay were performed to assess the stability of ARPC5. The interaction between CPEB2 and ARPC5 was confirmed by RNA immunoprecipitation assay.

**Results:**

CPEB2 and ARPC5 mRNA and protein expression levels were upregulated in CD138+ plasma cells from MM patients and cells. CPEB2 downregulation reduced MM cell proliferation, angiogenesis, and increased apoptosis, while its overexpression had an opposite effect. CPEB2 and ARPC5 were co-localized at cell cytoplasm and could positively regulate ARPC5 expression by mediating its mRNA stability. ARPC5 overexpression reversed the suppressive effect of CPEB2 knockdown on MM progression, and it knockdown also abolished CPEB2-promoted MM progression. Besides, CPEB2 silencing also reduced MM tumor growth by decreasing ARPC5 expression.

**Conclusion:**

Our results indicated that CPEB2 increased ARPC5 expression through promoting its mRNA stability, thereby accelerating MM malignant process.

## Introduction

Multiple myeloma (MM) is a blood disease characterized by clonal proliferation of malignant plasma cells in the bone marrow [1]. MM is considered to be an incurable hematologic malignancy, accounting for 10% of all hematologic tumors [2]. Although many effective anti-MM drugs have been developed with good results, most patients still relapse and have a poor prognosis [3, 4]. Therefore, there is an urgent need to elucidate the molecular mechanisms that influence MM progression.

RNA-binding proteins (RBPs) are important proteins in cells that regulate cell function by interacting with RNA [5, 6]. Cytoplasmic polyadenylation element binding protein (CPEB) family is a sequence-specific RBP consisting of five members, including CPEB1, CPEB2, CPEB3, CPEB4 and CPEB5. It is reported that CPEB1 is upregulated in MM cells and may participate in the pathogenesis of MM [7]. CPEB2 has been confirmed to play a key role in tumorigenesis [8]. Previous study suggested that CPEB2 acted as tumor promotor in endometrial carcinoma, which overexpression could accelerate cell proliferation and inhibit apoptosis [9]. Also, increased CPEB2 expression could promote the radio-resistance of breast cancer by enhancing cell growth [10]. In this, we detected high expression of CPEB2 in MM patients and cells. However, the role of CPEB2 in MM progression has not been studied.

Many studies have shown that CPEB family can regulate mRNA stability to mediate tumor progression [11]. For example, CPEB2 facilitated renal cancer cell proliferation and migration via decreasing p53 mRNA stability [12]. Actin-related protein 2/3 complex subunit 5 (ARPC5), a member of ARP2/3 complex family, is considered to be oncogene in the development of many tumors, such as lung squamous cell carcinoma [13] and hepatocellular carcinoma [14]. Studies had reported that ARPC5 was upregulated in MM patients and could predict the poor prognosis of MM patients [15]. Here, we found that CPEB2 could regulate ARPC5 mRNA stability. However, whether CPEB2 regulated ARPC5 mRNA stability to regulate MM malignant progression remains unclear.

In this research, we aimed to investigate CPEB2 roles in MM progression and its underlying molecular mechanism. We hypothesized that CPEB2 might promote the expression of ARPC5 by regulating its mRNA stability, and then accelerate the process of MM. Our study hopes to provide new insights into the deeper understanding of the pathogenesis of MM.

## Methods

### Samples

This study analyzed the CPEB2 and ARPC5 expression in the bone marrow samples collected from 20 MM patients who were treated at the First Affiliated Hospital, Department of Hematology, Hengyang Medical School, University of South China. Also, 20 healthy normal donors (iron deficiency anemia patients, due to menorrhagia or hemorrhoid blood loss, and excluded malignant tumors) were recruited in the First Affiliated Hospital, Department of Hematology, Hengyang Medical School, University of South China and their bone marrow samples were obtained. The percentage of CD138+ cells in the bone marrow of MM patients was obtained from the pathology report, and the purity of CD138+ plasma cells is over 90%. CD138 MicroBeads (Miltenyi Biotec GmbH, Bergisch Gladbach, Germany) were used to extract CD138+ primary plasma cells from the bone marrow samples of MM patients and healthy normal donors. Each subject signed written informed consent. This study was approved by the Ethics committee of the First Affiliated Hospital, Department of Hematology, Hengyang Medical School, University of South China.

### Cell culture

MM cell OPM2 was purchased from Biovector (Beijing, China), and RPMI-8226, NCl-H929, U266, MM1S and normal plasma cells (nPCs) were bought from ATCC (Manassas, VA, USA). Cells were cultured at 37 °C with 5% CO_2_ in RPMI-1640 medium (Gibco, Grand Island, NY, USA) containing 10% FBS (Gibco) and 1% penicillin/streptomycin (Invitrogen, Carlsbad, CA, USA).

### Quantitative real-time PCR (qRT-PCR)

RNA was isolated from cells using TRIzol reagent (Invitrogen), and cDNA was obtained by HiScript III RT SuperMix (Vazyme, Nanjing, China). Then, cDNA was amplified in PCR system by SYBR Green (Vazyme) with specific primers (Table 1). Gene expression was calculated 2^−ΔΔCt^ method with GAPDH as internal control.

**Table 1:** Primer sequences used for qRT-PCR

Gene	Forward (5′–3′)	Reverse (5′–3′)
CPEB2	GGAGCAACCATCAGAGCAGT	CCTGTAAGGGTAAGAGTGTATTACT
ARPC5	TGGTGTGGATCTCCTAATGAAGT	CACGAACAATGGACCCTACTC
GAPDH	CTCTGCTCCTCCTGTTCGAC	CGACCAAATCCGTTGACTCC

### Western blot

RIPA buffer (Beyotime, Shanghai, China) was used to extract total proteins. After quantified, protein was separated by 10% SDS-PAGE gel and transfer onto PVDF membranes. The membrane was blocked with nonfat milk, and incubated with anti-CPEB2 (ab222070, 1:100, Abcam, Cambridge, MA, USA), anti-ARPC5 (ab51243, 1:5000, Abcam) or anti-GAPDH (ab9485, 1:2500, Abcam). After hatched with secondary antibody (ab205718, 1:50,000, Abcam), membrane was treated with BeyoECL Moon (Beyotime) to observe protein signals. The gray value was analyzed by ImageJ software.

### Cell transfection

Lipofectamine 3000 (Invitrogen) was used to transfect with CPEB2 or ARPC5 lentivirus short hairpin RNA (sh-CPEB2 or sh-ARPC5), pcDNA overexpression vector (pc-CPEB2 or pc-ARPC5), and their negative controls (sh-NC and pc-NC) (synthesized by RiboBio, Guangzhou, China) into cells in-line with the manufacturer’s instructions.

### Cell counting kit 8 (CCK8) assay

MM cells were seeded into 96-well plates and cultured overnight. At the indicated time points, CCK8 solution (Beyotime) was used for incubation with cells for 4 h. Optical density (OD) values were analyzed using microplate reader at 450 nm.

### Soft-agar colony formation assay

MM cells suspended with 0.3% agar in complete medium were seeded in a 24-well plate containing complete medium with 0.5% agar. Cells were cultured for 2 weeks, and then the stained colonies were counted with AlphaView software.

### Flow cytometry

After transfection for 48 h, MM cells were harvested and suspended with binding buffer. After that cells were stained with Annexin V-FITC and propidium iodide using corresponding assay kit (Solarbio, Beijing, China). Apoptosis analysis was performed by flow cytometer (BD Bioscience, San Jose, CA, USA).

### Tube formation assay

The medium collected from transfected MM cells were harvested to prepare conditioned medium. HUVECs suspended with conditioned medium were seeded into 96-well plates pre-coated with Matrigel (BD Bioscience). Tube formation was observed after 8 h under a microscope and documented with ImageJ software.

### Fluorescence in situ hybridization (FISH) assay

OPM2 cells were fixed with 4% paraformaldehyde and then hybridized with FISH-CPEB2 probe and FISH-ARPC5 probe (Genepharma, Shanghai, China) overnight. After that cell nuclei was stained with DAPI, and fluorescence image was analyzed by confocal microscopy.

### Actinomycin D treatment

After transfection, MM cells were harvested with 20 μg/mL actinomycin D (Cell Signaling Technology, Danvers, MA, USA) as previously described [16]. At 0, 2, 4, 6 and 8 h post-reaction, cells were collected to measure ARPC5 mRNA expression by qRT-PCR.

### Cycloheximide (CHX) chase assay

Transfected MM cells were treated with 100 mM CHX (Millipore, Billerica, MA, USA) as previously described [11] for 0, 1, 3 and 6 h, respectively. At each incubation time, cells were used for protein extraction to detect ARPC5 protein expression using western blot analysis.

### RNA immunoprecipitation (RIP) assay

MM cells were transfected with pcDNA3.1(+)-CPEB2-Flag and incubated for 72 h. Then, cells were treated with RIP lysis buffer (Millipore), and cell lysates were pre-cleaned with protein G Sepharose beads. After incubated with anti-Flag antibody or anti-IgG antibody, the immunoprecipitated RNA was extracted for qRT-PCR to examine GAPDH and ARPC5 expression.

### Animal experiments

The BALB/c nude mice (Vital River, Beijing, China) were randomly divided into two groups: sh-NC group and sh-CPEB2 group (six mice in each group). OPM2 cells were transfected with sh-CPEB2/sh-NC, and then injected subcutaneously into mice. Tumor volume was measured every 7 d following injection. Mice were euthanized after 28 days and tumor tissues were collected. In addition to being weighed and examined, the tumor tissue was also made into paraffin sections for Ki-67 immunohistochemical (IHC) staining using anti-Ki-67 (ab15580, 1:200, Abcam) and SP Kit (Solarbio). Our study was approved by the Animal Ethics committee of the First Affiliated Hospital, Department of Hematology, Hengyang Medical School, University of South China.

### Statistical analysis

Data were presented as the mean ± SD and analyzed by Student’s *t* test (for two groups) or one-way ANOVA followed by Tukey post hoc test (for multiple groups). GraphPad 7.0 software was applied for data analysis. *P* < 0.05 was considered statistically significant.

## Results

### CPEB2 had elevated expression in MM patients and cells

**Fig. 1:**
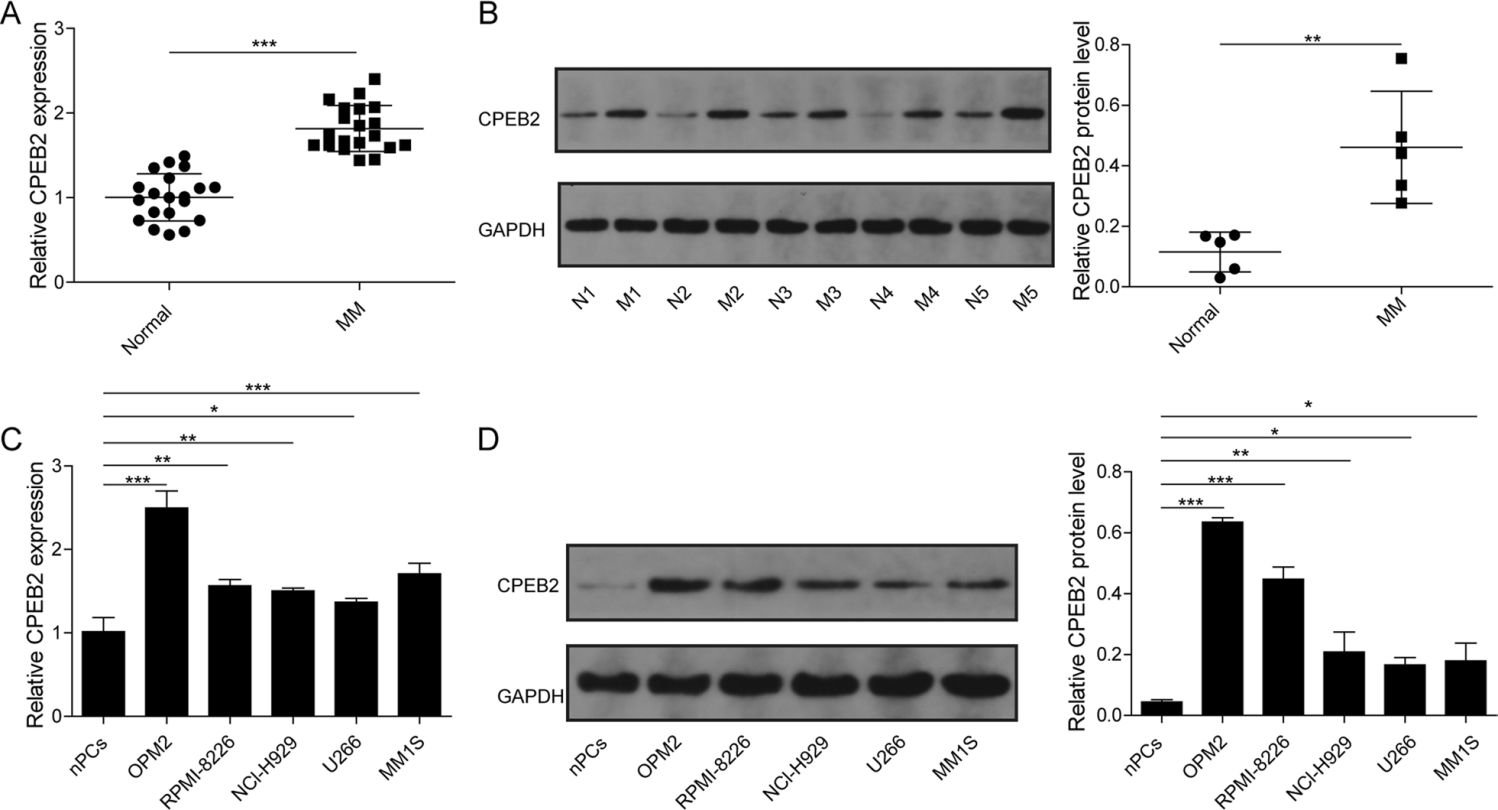
CPEB2 expression in MM patients and cells. **A** CPEB2 mRNA expression levels in the bone marrow CD138+ plasma cells from 20 MM patients and healthy normal donors was measured by qRT-PCR. **B** CPEB2 protein expression levels in the bone marrow CD138+ plasma cells from five MM patients (M1–M5) and five healthy normal donors (N1–N5) was measured by western blot. **C**, **D** qRT-PCR and western blot analysis were used to detect CPEB2 mRNA and protein expression in MM cells and nPCs cells. Cell experiment was repeated three times. Data were presented as the mean ± SD and analyzed by Student’s *t* test (for two groups) or one-way ANOVA followed by Tukey post hoc test (for multiple groups). *P < 0.05, **P < 0.01, ***P < 0.001

The expression of CPEB2 was detected by qRT-PCR and western blot analysis in the bone marrow CD138+ plasma cells of MM patients and healthy donors. The results showed that CPEB2 expression was markedly higher in MM patients than in healthy donors at the mRNA level and protein level (Fig. 1A, B). In addition, CPEB2 expression also was measured in MM cell lines and nPCs cells. As presents in Fig. 1C, D, CPEB2 mRNA and protein expression levels were significantly upregulated in five MM cell lines (OPM2, RPMI-8226, NCl-H929, U266 and MM1S). These data confirmed the high expression of CPEB2 in MM patients and cells.

### Knockdown of CPEB2 suppressed MM cell growth and angiogenesis

**Fig. 2:**
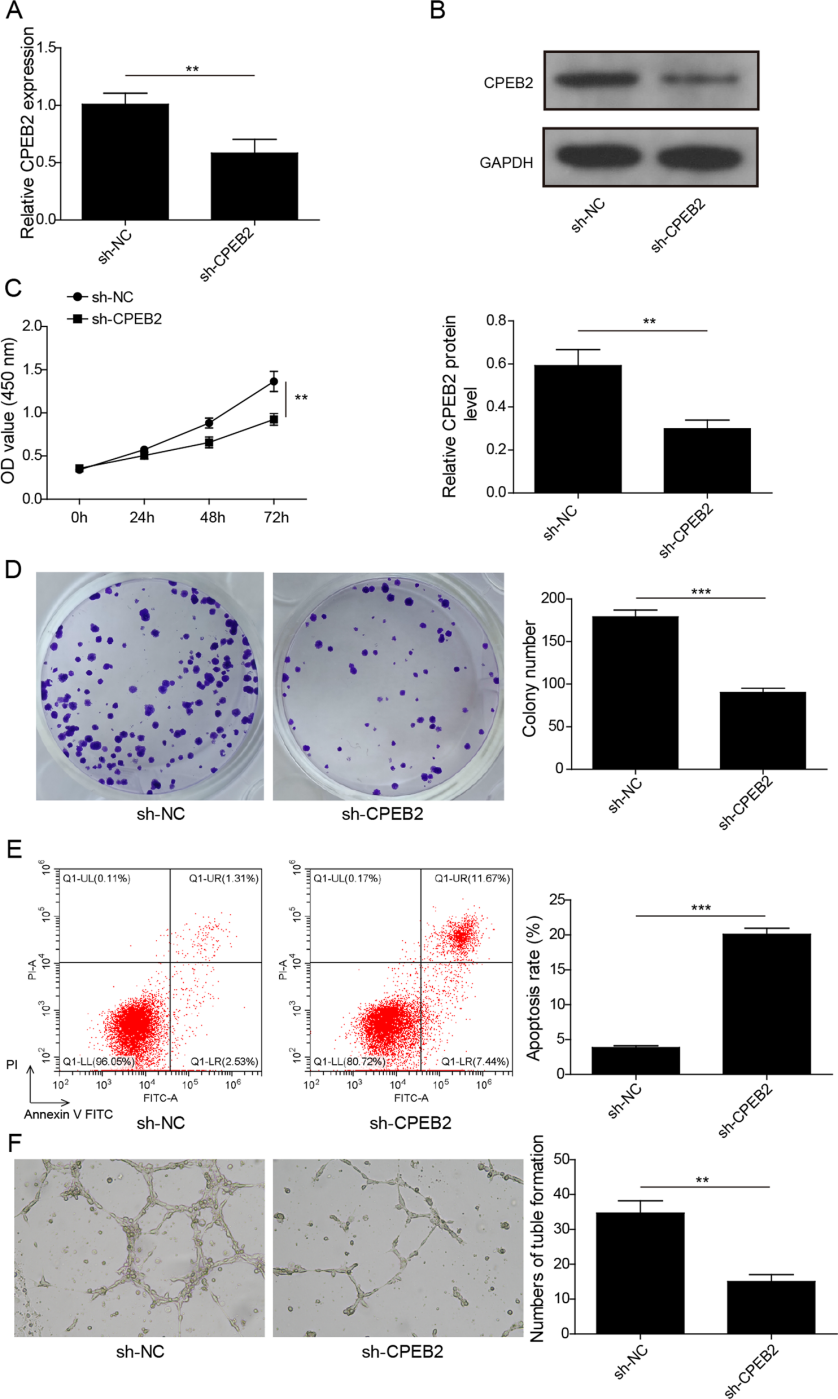
Effects of CPEB2 knockdown on MM cell growth and angiogenesis. OPM2 cells were transfected with sh-NC or sh-CPEB2. **A** CPEB2 mRNA expression was detected by qRT-PCR. **B** CPEB2 protein expression was detected by western blot. **C** CCK8 assay was performed to measure cell viability. **D** Soft-agar colony formation assay was used to detect colony number. **E** Flow cytometry was employed to measure cell apoptosis ratio. **F** Tube formation assay was used to assess angiogenesis ability. Cell experiment was repeated three times. Data were expressed as mean ± SD, and Student’s *t* test was used to compare data between two groups. **P < 0.01, ***P < 0.001

Next, loss-of-function experiments were performed to explore the role of CPEB2 in MM progression. After transfected with sh-CPEB2 into OPM2 cells, CPEB2 mRNA and protein expression levels were remarkably reduced (Fig. 2A, B). The results of CCK8 and soft-agar colony formation assay indicated that CPEB2 silencing reduced OPM2 cell viability and colony number (Fig. 2C, D). Furthermore, we observed that CPEB2 knockdown could increase the apoptosis ratio of OPM2 cells (Fig. 2E). Tube formation assay results indicated that downregulation of CPEB2 inhibited the number of tube formation (Fig. 2F). The above data showed that CPEB2 knockdown inhibited MM cell proliferation and angiogenesis, while enhanced apoptosis.

### Overexpression CPEB2 enhanced MM cell growth and angiogenesis

**Fig. 3:**
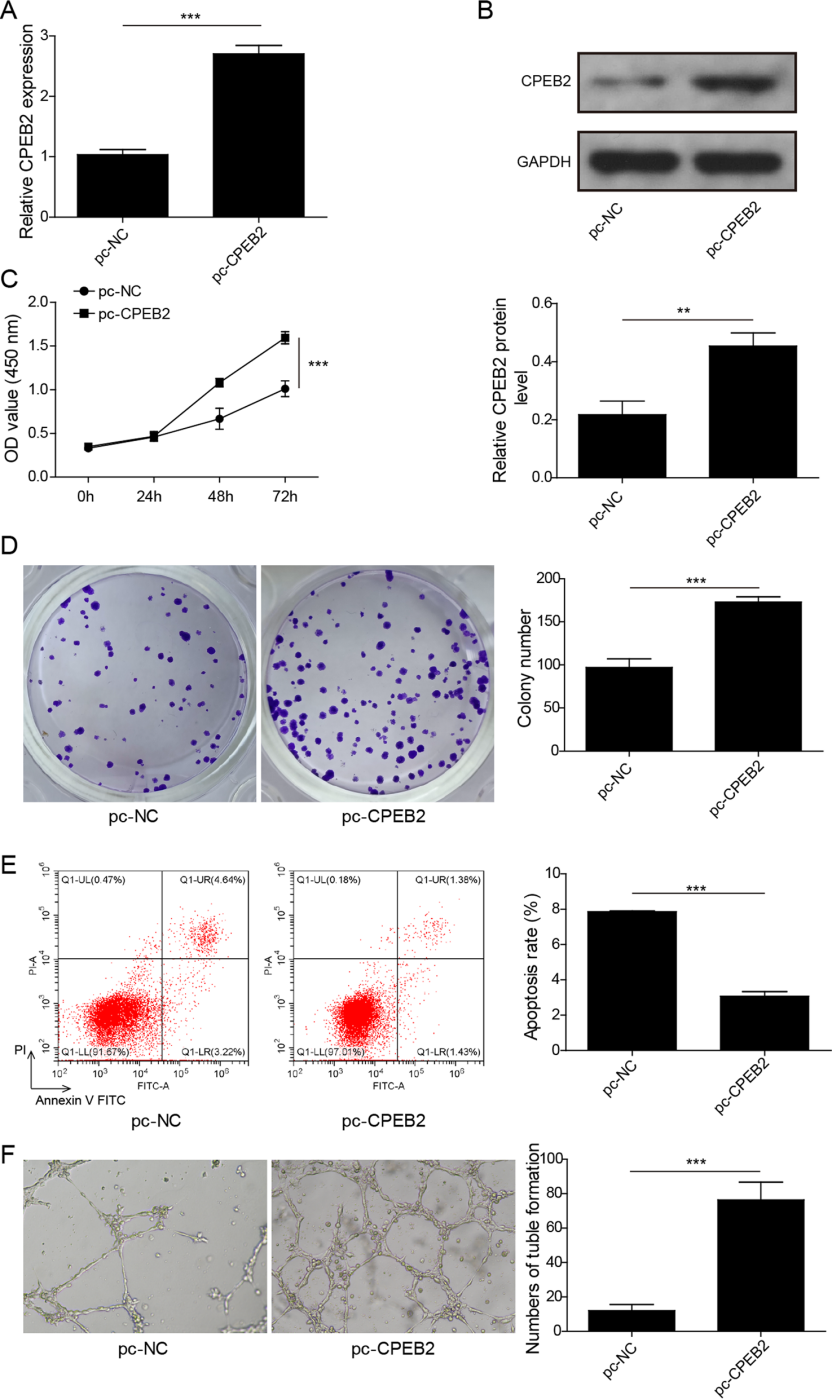
Effects of CPEB2 overexpression on MM cell growth and angiogenesis. U266 cells were transfected with pc-NC or pc-CPEB2. **A** CPEB2 mRNA expression was determined using qRT-PCR. **B** CPEB2 protein expression was determined using western blot. **C** Cell viability was detected by CCK8 assay. **D** Colony number was measured by soft-agar colony formation assay. **E** Cell apoptosis ratio was evaluated by flow cytometry. **F** Angiogenesis ability was assessed by tube formation assay. Cell experiment was repeated three times. Data were expressed as mean ± SD, and Student’s *t* test was used to compare data between two groups. **P < 0.01, ***P < 0.001

Additionally, pc-CPEB2 was transfected into U266 cells to overexpress CPEB2 at the mRNA and protein levels (Fig. 3A, B). Our data showed that CPEB2 upregulation not only promoted cell viability and colony number (Fig. 3C, D), but also inhibited cell apoptosis (Fig. 3E). Moreover, the number of tube formation also was enhanced by CPEB2 overexpression (Fig. 3F). These data confirmed that CPEB2 facilitated MM progression.

### CPEB2 increased ARPC5 expression by enhancing ARPC5 mRNA stability

**Fig. 4:**
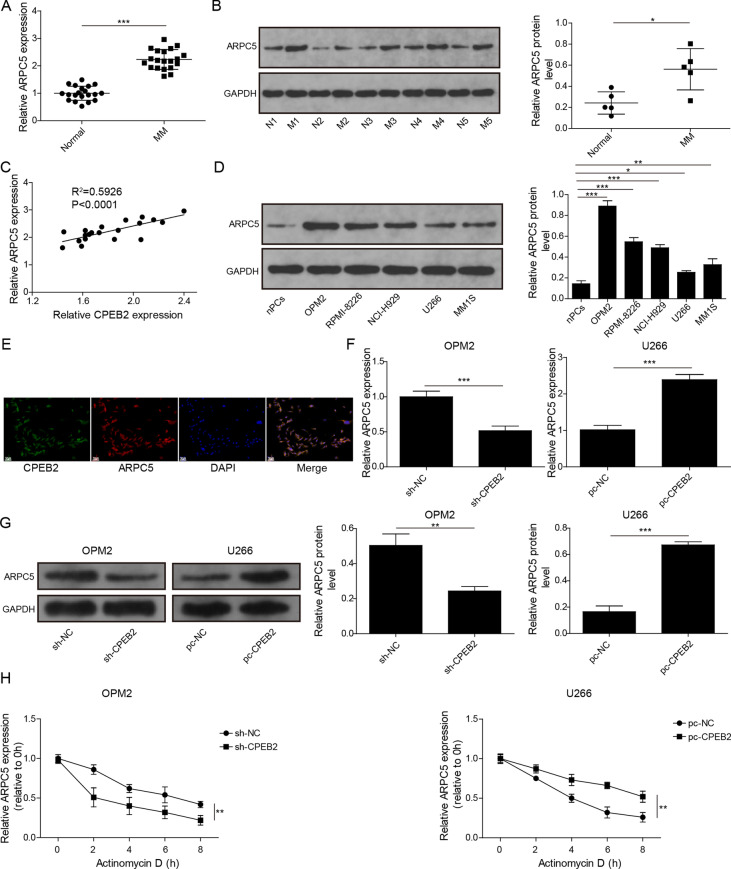

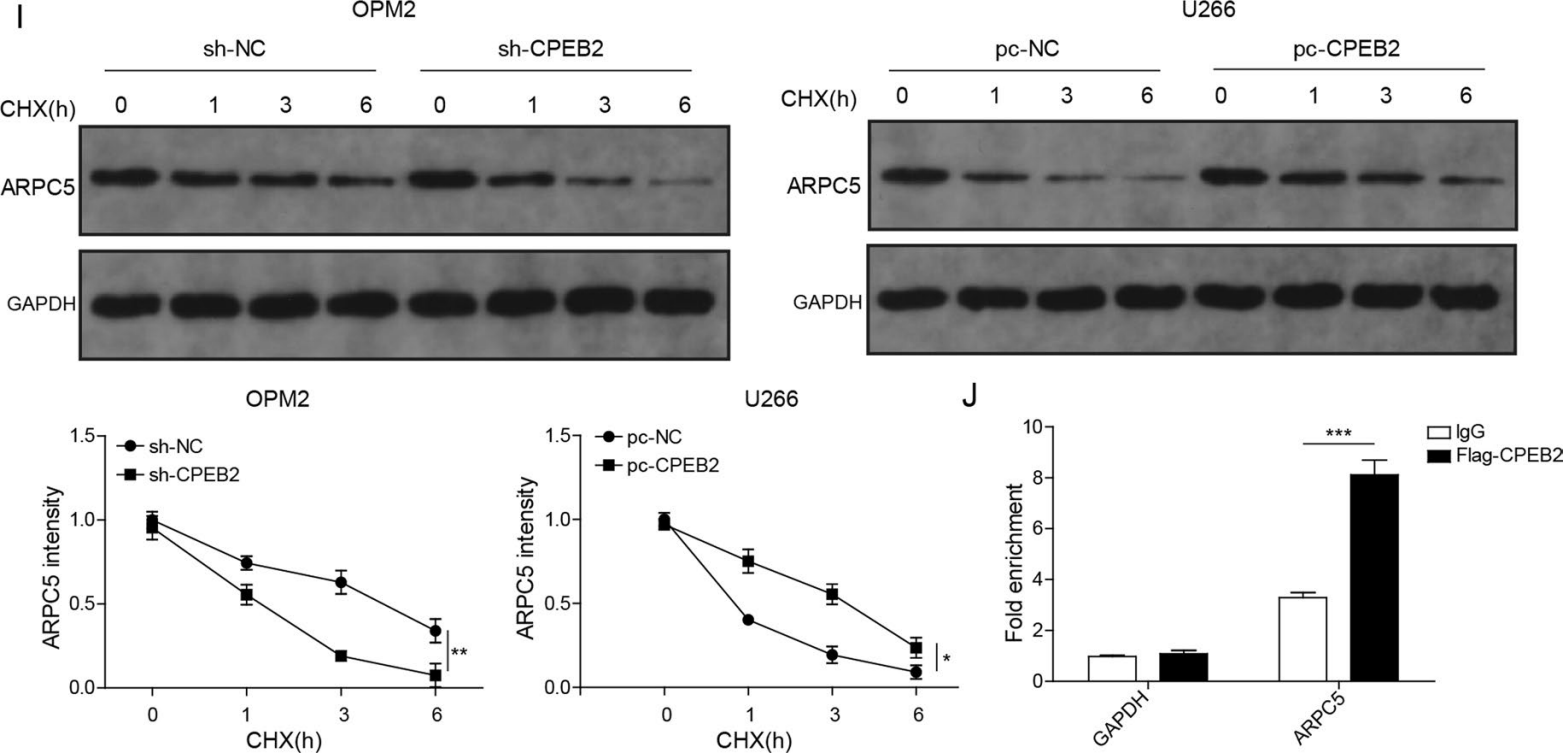
CPEB2 regulated ARPC5 expression. **A** ARPC5 mRNA expression levels in the bone marrow CD138+ plasma cells from 20 MM patients and healthy normal donors was measured by qRT-PCR. **B** ARPC5 protein expression levels in the bone marrow CD138+ plasma cells from five MM patients (M1–M5) and five healthy normal donors (N1–N5) was measured by western blot. **C** The correlation between CPEB2 and ARPC5 in MM was analyzed by Pearson method. **D** Western blot analysis was used to detect ARPC5 protein expression in MM cells and nPCs cells. **E** FISH assay was used to assess the co-localization of CPEB2 and ARPC5 in OPM2 cells. Scale bar, 50 μM. **F**, **G** ARPC5 mRNA and protein expression levels were examined by qRT-PCR and western blot analysis in OPM2 cells transfected with sh-CPEB2 or U266 cells transfected with pc-CPEB2. **H** Actinomycin D was used to assess the stability of ARPC5 in OPM2 cells transfected with sh-CPEB2 or U266 cells transfected with pc-CPEB2. **I** CHX treatment was performed to assess the effect of sh-CPEB2 or pc-CPEB2 on the half-life of ARPC5. **J** RIP assay was used to confirmed the interaction between CPEB2 and ARPC5 in U266 cells. Cell experiment was repeated three times. Data were presented as the mean ± SD and analyzed by Student’s *t* test (for two groups) or one-way ANOVA followed by Tukey post hoc test (for multiple groups). *P < 0.05, **P < 0.01, ***P < 0.001

Subsequently, we further explored the mechanism of CPEB2 regulating MM progression. Interestingly, the 3’-UTR of ARPC5 contains a CPE signal (UUUUUAU), suggesting that ARPC5 may be a potential target of CPEB2. Through qRT-PCR and western blot, we found that ARPC5 mRNA and protein levels were higher in the bone marrow CD138+ plasma cells of MM patients than that in healthy donors (Fig. 4A, B). Besides, Pearson correlation analysis showed that CPEB2 expression was positively correlated with ARPC5 expression in MM patients (Fig. 4C). Additionally, ARPC5 protein expression was upregulated in five MM cell lines (OPM2, RPMI-8226, NCl-H929, U266 and MM1S) compared to nPCs cells (Fig. 4D). FISH assay results suggested that CPEB2 and ARPC5 were co-localized in the cytoplasm of OPM2 cells (Fig. 4E). Then, we determined ARPC5 mRNA and protein expression under CPEB2 knockdown or overexpression in MM cells, and the results indicated that ARPC5 expression was decreased by CPEB2 knockdown and increased by CPEB2 overexpression (Fig. 4F, G). The stability of ARPC5 was detected by actinomycin D treatment under different transfection conditions. The data showed that CPEB2 knockdown could accelerate the degradation of ARPC5 mRNA, while its overexpression could stabilize ARPC5 mRNA (Fig. 4H). After CHX treatment, we found that CPEB2 knockdown resulted in the reduction of ARPC5 half-life, while CPEB2 overexpression prolonged the half-life of ARPC5 (Fig. 4I). RIP assay results showed that ARPC5 enrichment was increased in the Flag-CPEB2, indicating that CPEB2 could bind to ARPC5 transcripts (Fig. 4J). Above data revealed that CPEB2 could enhance ARPC5 mRNA stability to increase its expression.

### CPEB2 increased ARPC5 expression to promote MM cell growth and angiogenesis

**Fig. 5:**
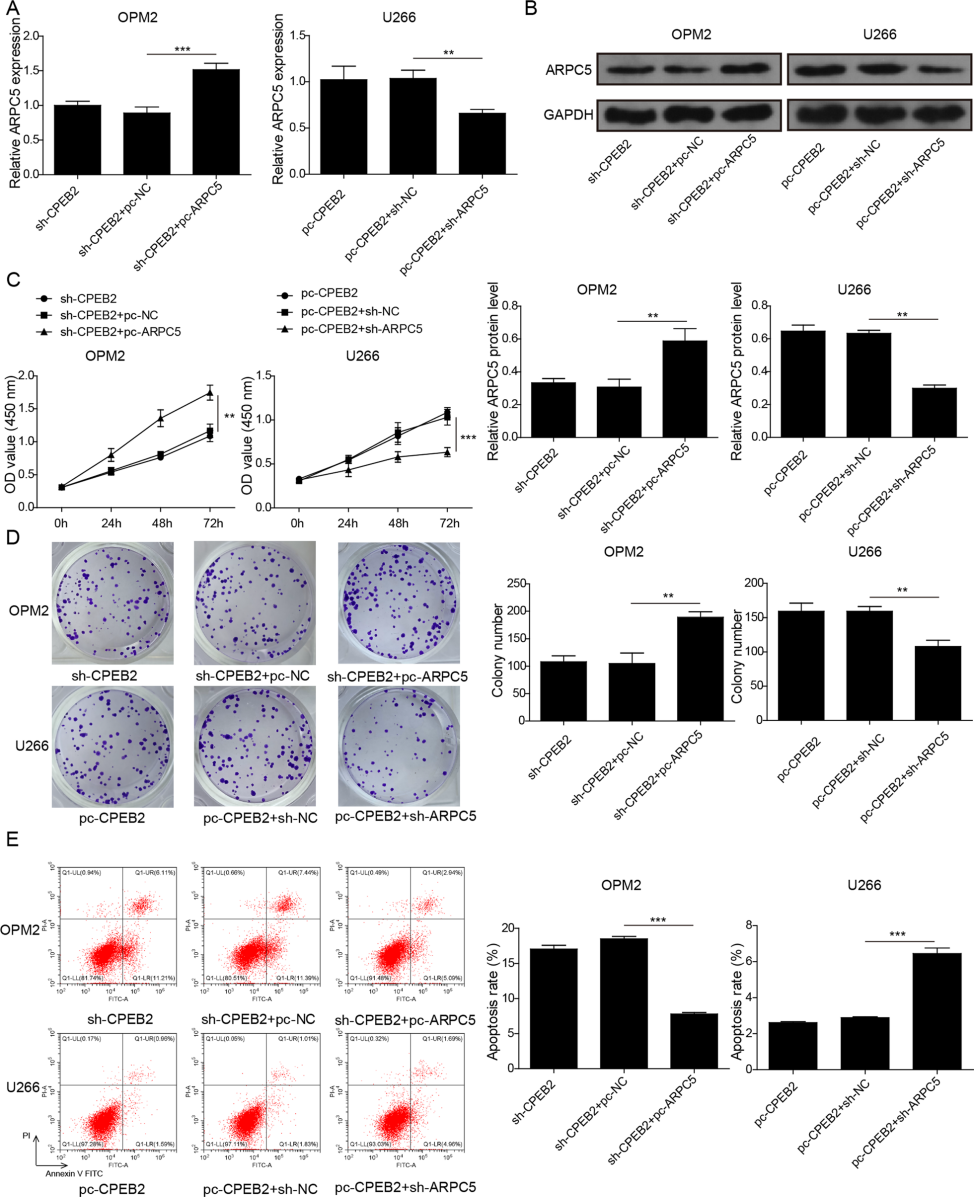

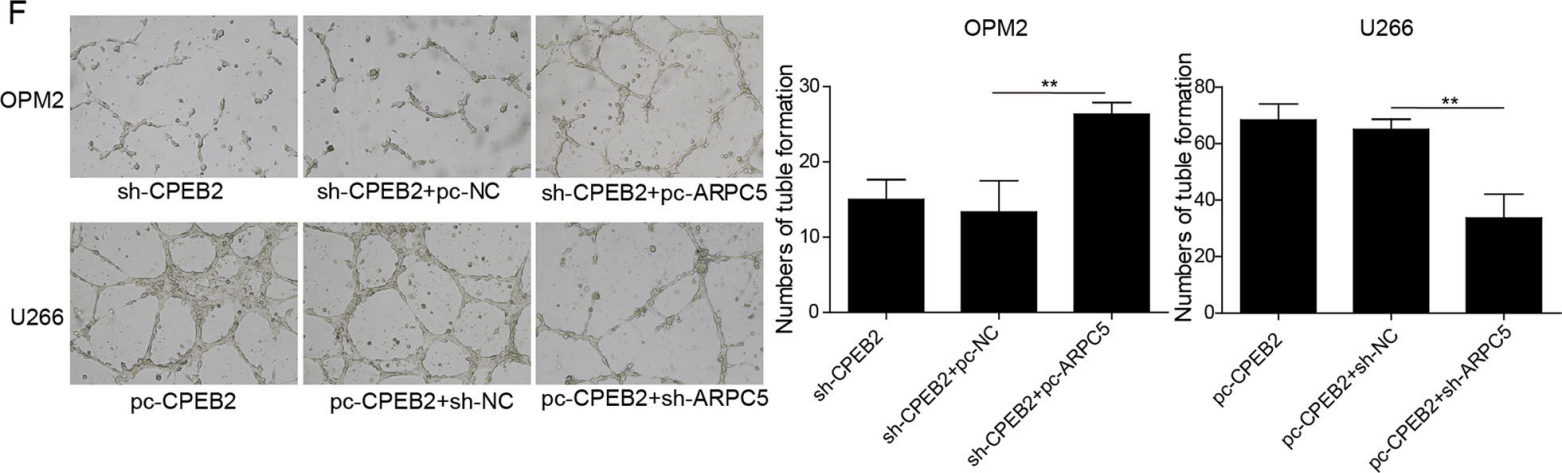
Effects of CPEB2 and ARPC5 on MM cell growth and angiogenesis. OPM2 cells were co-transfected with sh-CPEB2 and pc-ARPC5, and U266 cells were co-transfected with pc-CPEB2 and sh-ARPC5. **A**, **B** ARPC5 mRNA and protein expression levels were detected by qRT-PCR and western blot analysis. **C** CCK8 assay was used to detect cell viability. **D** Soft-agar colony formation assay was employed to examine colony number. **E** Cell apoptosis ratio was tested using flow cytometry. **F** Tube formation assay was performed to measure angiogenesis ability. Cell experiment was repeated three times. Data were expressed as mean ± SD, and one-way ANOVA followed by Tukey post hoc test was used to compare data between multiple groups. **P < 0.01, ***P < 0.001

To further confirm that CPEB2 promoted MM progression by increasing ARPC5 expression, we performed rescue experiments. OPM2 cells were co-transfected with sh-CPEB2 and pc-ARPC5, while U266 cells were co-transfected with pc-CPEB2 and sh-ARPC5. The detection results of ARPC5 mRNA and protein expression showed that the addition of pc-ARPC5 increased ARPC5 expression, and sh-ARPC5 reduced ARPC5 expression (Fig. 5A, B). Functional experiments showed that cell viability, colony number, the number of tube formation were enhanced, while apoptosis was repressed in the sh-CPEB2+pc-ARPC5 group compared to the sh-CPEB2+pc-NC group. On the contrary, cell viability, colony number, the number of tube formation were reduced, while apoptosis was promoted in the pc-CPEB2+sh-ARPC5 group compared to the pc-CPEB2+sh-NC group (Fig. 5C–F). All data fully confirmed that CPEB2 could increase ARPC5 expression, thereby facilitating MM cell growth and angiogenesis.

### Interference of CPEB2 inhibited MM tumor growth in vivo

**Fig. 6:**
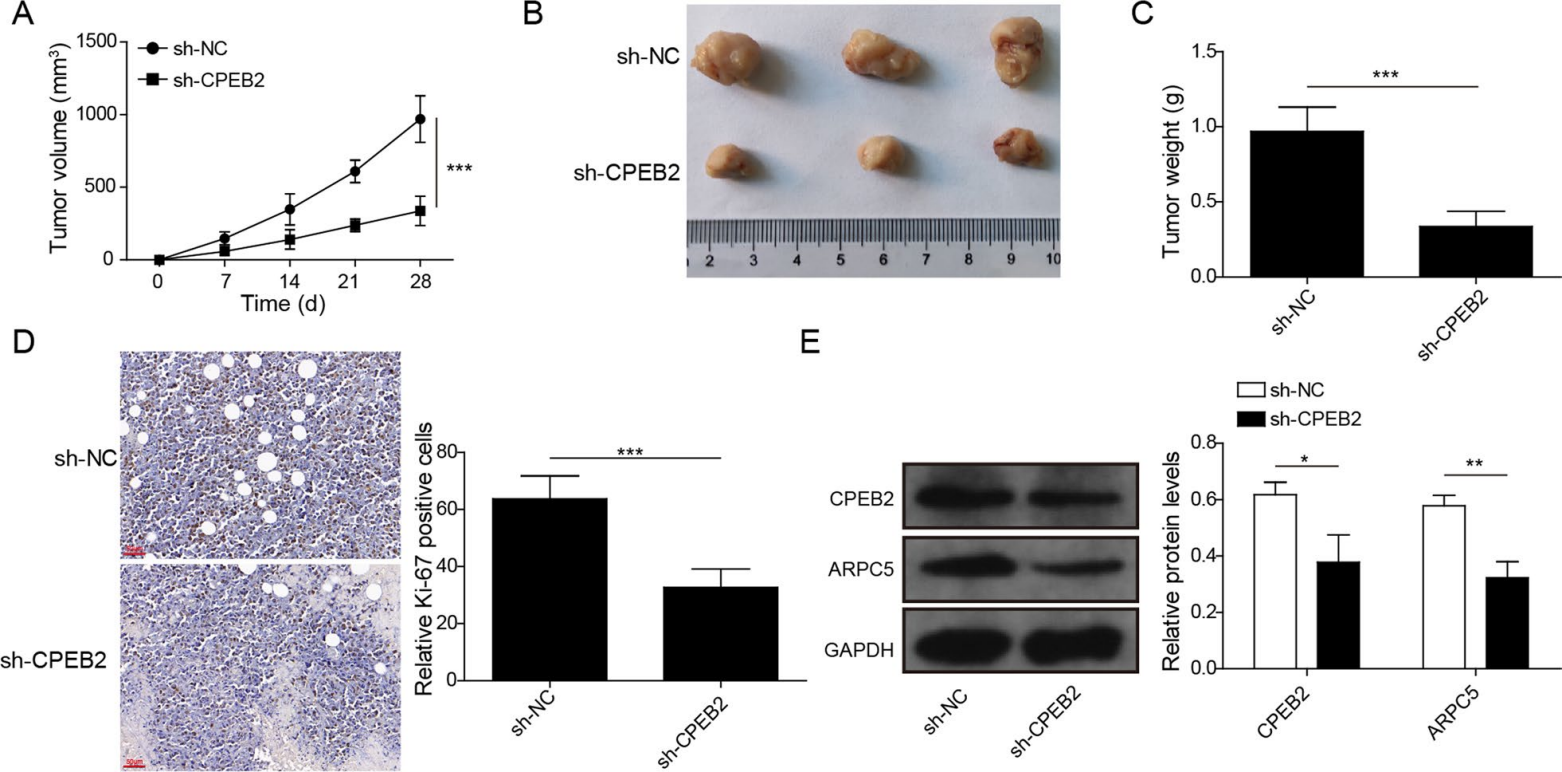
Effects of CPEB2 knockdown on MM tumor growth in vivo*.* OPM2 cells transfected with sh-NC or sh-CPEB2 were injected into mice. Tumor volume (**A**), size (**B**) and weight (**C**) were determined in each group. **D** IHC assay was used to assess Ki-67 positive cells in the tumor tissues of each group. Scale bar, 50 μM **E** CPEB2 and ARPC5 protein expression levels were determined by western blot analysis in the tumor tissues of each group. *n* = 6. Data were expressed as mean ± SD, and Student’s *t* test was used to compare data between two groups. *P < 0.05, **P < 0.01, ***P < 0.001

In order to further verify our results, we performed animal experiments. OPM2 cells stably transfected with sh-CPEB2 or sh-NC were injected into nude mice. After 28 days, we demonstrated a significant reduction in tumor volume, size, and weight in the sh-CPEB2 group (Fig. 6A–C). A low Ki-67 positive cells were found in the tumor tissues of the sh-CPEB2 group (Fig. 6D). Through western blot analysis, we confirmed that CPEB2 and ARPC5 protein expression levels were markedly decreased in the sh-CPEB2 group (Fig. 6E). These data illuminated that CPEB2 might promote MM tumor growth by regulating ARPC5 expression.

## Discussion

MM is the second most common hematological malignancy in the world, which is a serious threat to the health of patients [17]. Therefore, understanding the pathogenesis of MM is very important for the treatment of MM. RBPs are key participants in post-transcriptional events, and their mediated RNA regulatory network is closely related to tumorigenesis [18, 19]. Here, we investigated the role and potential mechanism of RBP CPEB2 in MM progression. Our study showed that CPEB2 upregulated the expression of ARPC5 by promoting its stability, and then facilitated the growth and angiogenesis of MM cells.

The role of CPEB2 in various tumor processes has been revealed. Wang et al*.* revealed that CPEB2 could accelerate paclitaxel resistance in nasopharyngeal cancer via increasing cell viability and decreasing apoptosis [20]. Besides, CPEB2 overexpression also could inhibit the methotrexate sensitivity of colorectal cancer [21]. Unfortunately, the role of CPEB2 in MM progression remains unclear. In this research, we observed that CPEB2 expression was upregulated in MM patients and cells, suggesting that CPEB2 might be involved in regulating MM progression. CPEB2 knockdown could effectively repress the proliferation and angiogenesis of MM cells, while promote its apoptosis. On the contrary, high expression of CPEB2 was able to enhance the process of MM cells, which was manifested as increased cell proliferation and angiogenesis ability, but decreased apoptosis. Moreover, the results of xenograft tumor experiments showed that interference of CPEB2 also reduced the tumorigenesis of MM in vivo. These results fully revealed that CPEB2 acted as an oncogene to mediate the progression of MM, and targeting CPEB2 might provide new ideas for MM treatment.

Importantly, studies had indicated that CPEB2 regulated the stability of many mRNAs, thereby affecting tumor progression. For example, CPEB2 reduces p53 mRNA stability to promote renal cancer cell progression [12], and, on the other hand, enhances SRSF5 mRNA stability to mediate glioma-specific chemotherapeutic effects [22]. Here, we pointed out that CPEB2 could elevate ARPC5 mRNA stability to mediate MM development. Similar to SRSF5, APRC5 might function as an oncogene via mRNA stability by CPEB2 interaction. ARPC5 is involved in actin synthesis and further influences the formation of microtubules, thus playing an important role in many cellular processes such as cell morphology maintenance [23, 24]. Several studies have shown that ARPC5 plays an important role in the development of tumors and can be used as a potential therapeutic target [14]. ARPC5 mediated melanoma cell migration and is critical for maintenance of YAP-dependent melanoma cell adhesion [25]. ARPC5 served as tumor promoter in head and neck squamous cell carcinoma, which silencing could restrain cell migration and invasion to hinder cancer process [26]. In addition, ARPC5 was verified to be upregulated in lung squamous cell carcinoma, and its knockdown significantly suppressed cell proliferation [13]. Based on microarray analysis, Xiong et al*.* revealed a high expression of ARPC5 in MM and determined that it was associated with poor tumor characteristics and survivals in MM patients [15]. This suggests that ARPC5 may be an important regulator of MM processes. Here, we demonstrated that ARPC5 was highly expressed in MM patients and cells, and CPEB2 upregulated ARPC5 expression by binding to its transcripts to increase its mRNA stability. Furthermore, we suggested that the pro-growth and pro-angiogenic ability of CPEB2 was dependent on the regulation of ARPC5 expression in MM. This is a new discovery for us.

Of course, there are still some limitations in this study. (1) We believe that the molecular mechanism of CPEB2 regulating MM progression should not only be this pathway, and ARPC5 is one of the downstream target of CPEB2. Whether CPEB2 mediates other molecules to regulate MM progression will be the focus of our future research. (2) Previous studies have reported that ARPC5 may affect MM cell proliferation through mTORC1 pathway using gene set enrichment analysis [15]. However, whether ARPC5 affects MM processes by regulating the mTORC1 pathway remains to be further verified. Therefore, whether mTORC1 signaling pathway is involved in the regulation of CPEB2/ARPC5 axis on MM progression is the focus of our subsequent research. (3) In this study, although we points out that CPEB2 expression is elevated in MM patients, we have not yet analyzed the relationship between CPEB2 expression and the prognosis of MM patients. In the future, more MM patients should be included, and clinical data should be collected to analyze the relationship between CPEB2 expression and the prognosis of MM patients, so as to provide evidence that CPEB2 may be a potential biomarker of MM prognosis.

## Conclusions

In summary, our study revealed that CPEB2 promoted MM cell growth and angiogenesis by increasing ARPC5 mRNA stability to upregulate its expression. Therefore, this study highlights the potential mechanism by which CPEB2 facilitated MM progression and provides a new promising molecular target for MM treatment.

## Data Availability

All data generated or analyzed during this study are included in this published article.
